# Renal Failure Associated with APECED and Terminal 4q Deletion: Evidence of Autoimmune Nephropathy

**DOI:** 10.1155/2010/586342

**Published:** 2010-12-14

**Authors:** Mohammed Al-Owain, Namik Kaya, Hamad Al-Zaidan, Ibrahim Bin Hussain, Hadeel Al-Manea, Hindi Al-Hindi, Shelley Kennedy, M. Anwar Iqbal, Hamad Al-Mojalli, Albandary Al-Bakheet, Anne Puel, Jean-Laurent Casanova, Saleh Al-Muhsen

**Affiliations:** ^1^Department of Medical Genetics, King Faisal Specialist Hospital and Research Centre, Riyadh 11211, Saudi Arabia; ^2^College of Medicine, Alfaisal University, Riyadh 11533, Saudi Arabia; ^3^Department of Genetics, King Faisal Specialist Hospital and Research Centre, Riyadh 11211, Saudi Arabia; ^4^Department of Pediatrics, King Faisal Specialist Hospital and Research Centre, Riyadh 11211, Saudi Arabia; ^5^Department of Pathology and Laboratory Medicine, King Faisal Specialist Hospital and Research Centre, Riyadh 11211, Saudi Arabia; ^6^Ontario Newborn Screening Program, Children's Hospital of Eastern Ontario, Ottawa, ON, Canada K1H8L1; ^7^Pathology and Laboratory Medicine, University of Rochester Medical Center, Rochester, NY 14642, USA; ^8^Laboratory of Human Genetics of Infectious Diseases, Necker Branch, Inserm U550, 75015 Paris, France; ^9^Department of Pediatrics, College of Medicine and Prince Naif Center for Immunology Research, King Saud University, P.O. Box 2925, Riyadh 11461, Saudi Arabia; ^10^Department of Pediatrics, College of Medicine, King Saud University, Riyadh 11461, Saudi Arabia

## Abstract

Autoimmune polyendocrinopathy-candidiasis-ectodermal dystrophy (APECED) is a rare autosomal recessive disorder caused by mutations in the autoimmune regulator gene (*AIRE*). Terminal 4q deletion is also a rare cytogenetic abnormality that causes a variable syndrome of dysmorphic features, mental retardation, growth retardation, and heart and limb defects. We report a 12-year-old Saudi boy with mucocutaneous candidiasis, hypoparathyroidism, and adrenocortical failure consistent with APECED. In addition, he has dysmorphic facial features, growth retardation, and severe global developmental delay. Patient had late development of chronic renal failure. The blastogenesis revealed depressed lymphocytes' response to *Candida albicans* at 38% when compared to control. Chromosome analysis of the patient revealed 46,XY,del(4)(q33). FISH using a 4p/4q subtelomere DNA probe assay confirmed the deletion of qter subtelomere on chromosome 4. Parental chromosomes were normal. The deleted array was further defined using array CGH. *AIRE* full gene sequencing revealed a homozygous mutation namely 845_846insC. Renal biopsy revealed chronic interstitial nephritis with advanced fibrosis. In addition, there was mesangial deposition of C3, C1q, and IgM. This is, to the best of our knowledge, the first paper showing evidence of autoimmune nephropathy by renal immunofluorescence in a patient with APECED and terminal 4q deletion.

## 1. Introduction

*AIRE* gene was cloned in 1997 by two independent groups [1, 2]. Mutations in the *AIRE* cause autoimmune polyendocrinopathy-candidiasis-ectodermal dystrophy (APECED, OMIM no. 240300), also known as autoimmune polyglandular syndrome type 1. APECED is diagnosed based on the presence of two of a triad: hypoparathyroidism, adrenocortical failure, and chronic mucocutaneous candidiasis [3, 4]. APECED has a high prevalence among Finns (1/25000), Sardinians (1/14000), and Iranian Jews (1/8000). Several other disorders are part of APECED like pernicious anemia, vitiligo, thyropathy, gonadal failure, diabetes mellitus, and autoimmune hepatitis [4–6]. There are also reports of chronic interstitial nephritis in patients with APECED [7, 8]. *AIRE* clearly plays a crucial role in preventing organ-specific autoimmunity. It regulates the expression of ectopic proteins expressed by medullary thymic epithelial cells which contribute significantly to central tolerance; thus preventing autoimmunity and production of autoantibodies, and elucidating the significant autoimmune manifestations in APECED and *AIRE^−/−^* mice [5, 9, 10]. 

The terminal deletion of 4q results in a recognizable syndrome. The deletion 4q33 has been described in 15 patients; most of these cases presented with craniofacial anomalies, mental retardation, poor growth, and variable heart and limb defects [11–15]. It seems that severity of the phenotype correlates with the size of the deletion ranging from mild physical signs in 4q34 deletion to more severe phenotype in deletion involving 4q31 [12, 15–20]. It appears that 4q33 is the critical region of the 4q terminal deletion syndrome [19]. Renal disease in the form of absorptive hypercalciuria and kidney calcification has been reported in few children with terminal 4q deletion [13, 21]. 

Herein, we describe a patient with terminal deletion of 4q and features consistent with APECED. In addition, he developed autoimmune renal involvement leading to renal failure.

## 2. Patient and Diagnosis

The study was approved by the Research Advisory Council at King Faisal Specialist Hospital and Research Centre, Riyadh, Saudi Arabia (RAC no. 2040042). Written informed consent was given by the parents. 

### 2.1. Patient Report

**Figure 1:**
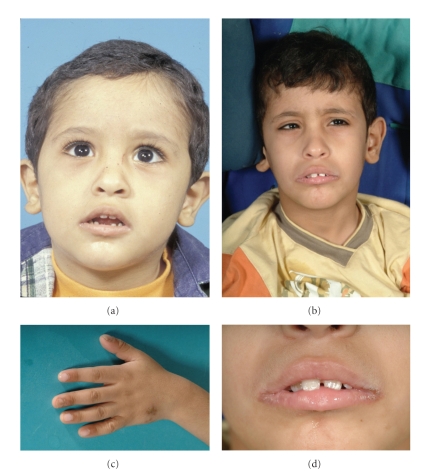
Photograph of the patient showing subtle dysmorphic features with high forehead, flat facies, large protruding ears, and large mouth, at the age of 7 years (a) and at the age of 9 years (b). Fungal infection (*Candida albicans*) of the nails (thumb and middle finger) and dorsum of the hand (c) and around the mouth corners (d).

**Figure 2:**
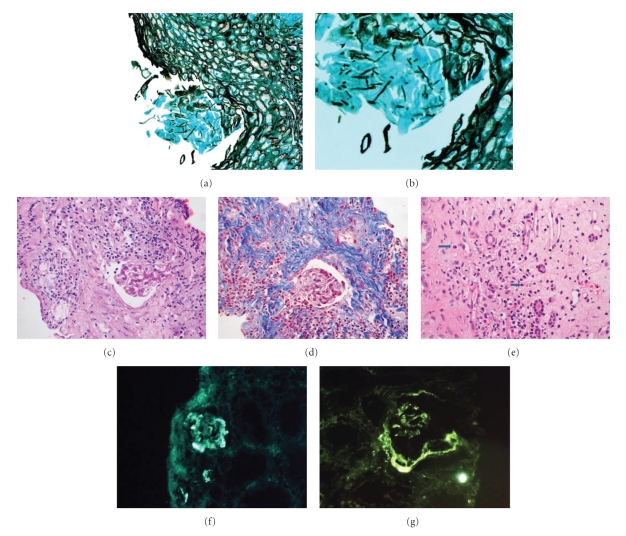
Superficial esophageal mucosa ((a),(b)) invaded by candidal psudohyphae. Grocott-Methenamine silver (original magnification 200x (a)/400x (b)). Renal biopsy ((c), (d), (e)) shows ischemic glomerular tuft with thick and wrinkled basement membrane (PAS stain 40x) (c) and extensive tubular loss with replacement by fibrosis (Masson trichrome stain 40x) (d). The interstitium shows lymphocytes and plasma cells (arrows) (H&E 40x) (e). The immunofluorescence staining for C3 and C1q is shown in (f) and (g), respectively.

The patient is a 12-year-old Saudi boy (Figure 1) who was brought initially to medical care at the age of 6 months because of delayed developmental milestones. Both parents are reported to be healthy. They are consanguineous (1st cousins). They have 5 other children, 4 girls and 1 boy, who are all reported to be healthy. The patient was born at term to a 38-year-old mother and a 39-year-old father following an uncomplicated pregnancy. At 18 months of age, the patient was noted to have oral lesions and nail dystrophy (Figures 1(c) and 1(d)). Cultures grew *Candida albicans*. His medical history was negative for recurrent chest infections, skin abscesses, or chronic diarrhea. Nitro Blue Tetrazolium (NBT), leukocytic markers, and immunoglobulin levels were normal. HIV test was negative. The blastogenesis revealed depressed lymphocytes' response to candida at 38% when compared to control. Nonetheless, it gave a robust response to mitogens and other antigens. He was treated with oral fluconazole. Because of recurrent vomiting, an upper GI Endoscopy was performed and revealed candida esophagitis (Figures 2(a) and 2(b)). The presence of diffuse cerebellar atrophy was noted on followup MRI. Developmentally, the patient had global delays. He sat at 1 year and stood with support at 2 years. Bayley Scales of Infant Development when the patient was 2.5 years old revealed a mental age of 11 months and a motor age of 6 months. Review of the family history was negative for recognized genetic conditions, congenital anomalies, and mental retardation. The patient is followed in ophthalmology for esotropia and horizontal nystagmus. His weight and height at the age of 8 years were less than the 3rd centile, and his head circumference was 51 cm, on the 25th centile. He was subtly dysmorphic with a mask-like facies, high forehead, epicanthal folds, thin upper lip, and smooth philtrum, large mouth, and crowded teeth. On CNS exam, he was noted to have increased tone, deep tendon reflexes at 3/4, and downward flexed feet. He had sometimes abnormal involuntary and purposeless athetoid-like movement of the upper extremities. Since the age of 8 years, the renal function has been noticed to gradually decline with the urea rising from 4.8 to 35.2 mmol/L, and creatinine climbing from 67 to as high as of 1223 *μ*mol/L. The renal ultrasonography showed small kidneys consistent with chronic renal failure. The patient did not have evidence of absorptive hypercalciuria.

## 3. Materials and Methods

### 3.1. Cytogenetics and FISH Analysis

Cytogenetic analysis was performed from phytohemagglutinin stimulated lymphocyte cultures by routine laboratory protocol. For microscopic analysis, metaphase chromosomes were stained with trypsin-Giemsa technique, and 20 cells were analyzed and two to five metaphases were karyotyped. Chromosome 4ToTelVysion multicolor subtelomere DNA probes mixture; 4PTEL02 labelled with spectrum green and AFMA224XH1 (4Q subtelomere) labelled with spectrum orange was used to hybridize the p and q telomeres of chromosome 4 pair. Pre- and posthybridization procedure was performed as per manufacturer's recommendation (Abbot-Vysis Inc., Catalog no. 33-270000). At least 5–10 metaphases were analyzed if possible at 100x magnification objective under oil immersion.

### 3.2. Array CGH Experiments and Data Analysis

We used the Human Genome CGH Microarray Kit 244A (Agilent Technologies, Santa Clara, CA, USA) according to the manufacturer's instructions with minor modifications. The Bioprime Labeling Kit (Invitrogen Inc., Carlsbad, CA, USA) was used to label all of digested patient's DNA and reference DNA separately with Cy5-dUTP and Cy3-dUTP (PerkinElmer, Waltham, MA, USA), respectively. Labelled patient DNA and reference DNA were mixed, purified, and prepared for hybridization. After 38 hrs of hybridization, repeated wash cycles were performed according to the manufacturer's instructions. The images and data generated by the feature extraction tool were loaded into CGH Analytics 3.4.40 software (Agilent Technologies) to allow visualization of the data.

### 3.3. AIRE Gene Sequencing

Genomic DNA was isolated by phenol/chloroform extraction. The 14 *AIRE* exons were PCR-amplified (with or without 7.5% of DMSO), sequenced, and analyzed on an ABI Prism 3700 apparatus (BigDye Terminator sequencing kit, Applied Biosystems). The primers used for PCR and sequencing are provided in Table 1.

### 3.4. Renal Histology and Immunofluorescence

Fresh core renal biopsy was examined under dissecting microscope for the presence of cortex with glomeruli. Once cortex was identified, a small (1 mm) fragment was frozen for immunofluorescence. The remaining tissue was fixed in 10% formalin for routine histology. Sections (2-3 *μ*m) were prepared and stained with H&E (hematoxylin and eosin stain), PAS (Periodic acid-Schiff), Jones', Masson trichrome, and Congo red stains. Primary antibodies directly conjugated to fluorescein isothiocyanate were utilized. No secondary antibodies were used.

## 4. Results

### 4.1. Hormonal and Autoantibodies Assays

During followup, the patient was investigated for associated polyendocrinopathy and found to have hypoparathyroidism and adrenal insufficiency with markedly low parathyroid hormone (PTH) despite impaired renal function. The PTH was at 3-4 (15–65 ng/L), the adrenocorticotropic hormone (ACTH) was high at 75–129 (5–60 ng/L), and the free T4 was slightly elevated at 24-25 (12–22 pmol/L) while the TSH was normal. In addition, the blood glucose and hemoglobin A1c were normal. The antinuclear antibody (ANA) screen and quantitative ANA were negative. A host of autoantibodies including antiplatelet, anti HLA class I, antithyroid Peroxidase, antithyroglobulin, liver and kidney microsomal antiglomerular basement membrane, antimitochondrial, and intrinsic factor autoantibodies were all normal. The antismooth muscle autoantibodies were weakly positive. The antiproximal tubular autoantibody testing was not available.

### 4.2. Renal Biopsy

The renal biopsy (Figures 2(c), 2(d), and 2(e)) showed chronic tubular interstitial nephritis with advanced fibrosis. The immunofluorescence demonstrated mesangial deposition of C3 (3+), C1q (2+) (Figures 2(f) and 2(g)), and IgM (1+) (figure not shown) in the glomerulus examined. The remaining immunoglobulins were negative in the glomerular basement membrane, mesangium, and extraglomerular structures.

### 4.3. Cytogenetic Analysis and FISH

**Figure 3:**
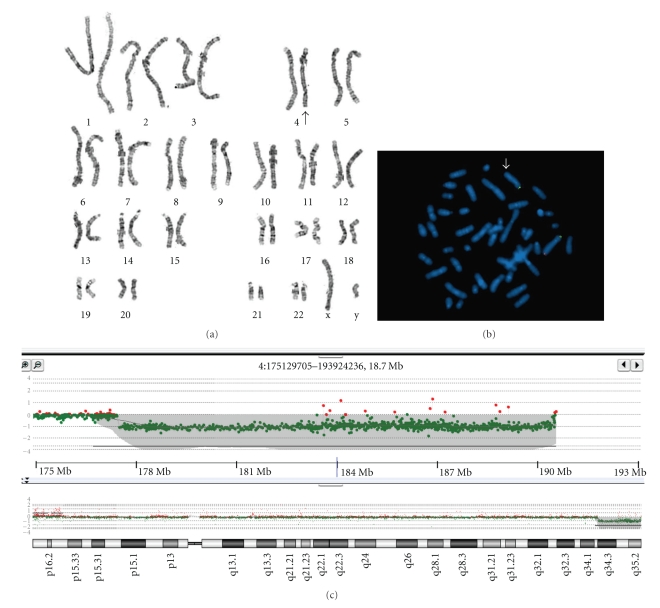
Trypsin-Giemsa karyotype 46,XY,del(4)(q33) from peripheral blood cells in the patient, the arrow indicates the abnormal chromosome 4 (a). Metaphase FISH image (b) from peripheral blood hybridized with Vysis 4p/4q subtelomere probes. The small arrow shows the deletion of the 4qsubtel region (red) on the abnormal 4q33 deletion chromosome. The 4psubtel region shows the normal green signals for both chromosomes 4. ish del(4)(q33)(4psubtel+; 4qsubtel−). The array CGH (c) depicting the terminal deletion on long arm of chromosome 4 and its breakpoints. The deletion starts at 177,732,852 and ends at 191,279,059 bp on Chr4 and is nearly 13.5 Mb. There are 72 genes in the region based on NCBI Build 35.1 for *Homosapiens*.

The chromosomal study revealed 46,XY,del(4)(q33) (Figure 3(a)). Parental chromosomes were normal. Fluorescence in situ hybridization (FISH) analysis using the 4P/4Q subtelomere DNA probe confirmed the presence of deletion 4q33 (Figure 3(b)).

### 4.4. Array CGH

The array CGH (Figure 3(c)) showed a large deletion that starts at 177,732,852 and ends at 191,279,059 bp on chromosome 4 and refined the terminal deletion to be starting at 4q34.3. The size of the deletion is nearly 13.5 Mb and has 90 genes in the region based on NCBI Build 37.1 for *Homosapiens *(supplementary table available online at doi: 10.1155/2011/586342).

### 4.5. AIRE Gene Sequencing

A c.845_846insC mutation (p.Leu283SerfsX6) in exon 7 was found in the *AIRE* gene.

## 5. Discussion

APECED is a monogenic disease with autosomal recessive inheritance characterized by multiple organ-specific autoimmunity. The *AIRE* gene encompasses 13 kb of genomic DNA and includes 14 exons. It encodes a 545-amin-acid transcription regulator of 57.5 kDa. To date, more than 50 mutations have been identified in the coding region [5, 22]. Pathogenic mutations within the *AIRE* gene trigger an autoimmune destructive process in the target organs by disturbing the immunological tolerance of the patients [23]. Our patient has interesting co-occurrence of *AIRE* mutation causing APECED and the 4q terminal deletion syndrome. A single patient report of renal failure was reported in APECED in whom the renal biopsy revealed tubulointerstitial nephritis with severe glomerular sclerosis, but no renal immunofluorescence result was mentioned. Interestingly, multiple acute rejection episodes occurred after renal transplant, and chronic rejection resulted in lost graft that required retransplantation. The patient had a large homozygous deletion (g.424_2157del1734) in *AIRE* gene. Antiproximal tubular autoantibodies were detected in the patient, and no other etiology of chronic interstitial nephritis was found, suggesting that the autoimmune mechanism was important in the development of the interstitial nephritis [8]. Tubulointerstitial nephritis was reported in 9% of the Finnish APECED patients, the largest international series [7]. The renal biopsy in our case confirms the same findings of the tubulointerstitial involvement reported by Ulinski et al. [8]. In our patient, however, the presence of C1q and C3 strong mesangial deposition in the glomerulus examined supports the evidence of autoimmune nephritis as the cause of the renal failure. The homozygous base pair insertion of cytosine at position 845 in our patient leads to a frame shift and a stop codon at amino acid 288 of the AIRE protein. It was previously reported only in one patient from Saudi Arabia who has APECED without renal involvement [24]. It has been hypothesized that renal autoantibodies could play a role in the initial development of chronic interstitial nephritis [8]. 

In APECED, autoantibodies are wellknown to be directed against various tissues (e.g., adrenal glands, liver, gonads, and thyroid). On the cellular level, several autoantibodies are directed against the cytochrome P450 enzyme (CYP) system like 17 a hydroxylase (CYPC17), 21-hydroxylase (CYP21) but may target other antigens such as tryptophan hydroxylase (TPH), tyrosine hydroxylase, interferons, and interleukin 17 and 22 cytokines, among others [4, 25–28]. Furthermore, SOX9 and SOX10 transcription factors are vitiligo autoantigens in APECED [29]. Association between the specific autoantibodies and the disease process in APECED has been established in Addison's disease, hypoparathyroidism, hypogonadism, diabetes mellitus, vitiligo autoimmune hepatitis, intestinal dysfunction, pernicious anemia, alopecia, and autoimmune thyroid disease [4, 9, 30]. 

Our patient has the craniofacial features of 4q deletion syndrome, as well as the profound psychomotor retardation and poor growth. To the best of our knowledge, renal anomalies have not been reported in 4q33 terminal deletion cases. The only patient reported by Strehle and Bantock [15] to have renal failure and 4q- syndrome had an interstitial deletion 4q32q35. The patient underwent an emergency cardiac surgery and developed kidney failure in the neonatal period (personal communication). The area deleted in our patient harbors a large number of genes, many of which are of unknown function or have not been linked to a specific phenotype. A gene of interest in the region deleted is the toll-like receptor 3 gene (TLR3) at 4q35. Toll-like receptors (TLRs) play an important role as a critical link between the innate and adaptive immunity, and a disregulated TLR signaling may be associated with autoimmune diseases [31]. The role of haploinsufficiency of TLR3 remains to be elucidated. A dominant-negative TLR3 allele in otherwise healthy children with herpes simplex virus 1 encephalitis was reported. Mutations in TLR3 impair TLR3 responses and confer a predisposition to herpes simplex encephalitis [32]. 

The primary cause of the development of autoimmune interstitial nephritis is quite intriguing in our patient including the *AIRE* mutation, the 4q deletion, and possibly some unknown genetic factor due to consanguinity. *AIRE* gene defect may be favored as significantly contributing to the autoimmune nephropathy based on the following. (i) The autoimmune nature of the renal involvement in our patient based on renal biopsy immunofluorescence. (ii) The pathogenesis in APECED is based on autoimmunity, with well-described multiorgan autoimmune dysfunction. (iii) A previous case with renal failure and APECED and positive antiproximal tubular autoantibodies. (iv) Lack of reported cases with 4q terminal deletion syndrome and renal failure due to tubulointerstitial nephritis. Nevertheless, this also might be the first case of autoimmune interstitial nephritis because of terminal 4q deletion since the number of all reported cases is small, and some form of renal involvement in terminal 4q deletion was previously reported. In addition, it is difficult to know whether the deletion of TLR3 or some other 4q genes had any contributory effect on the development of autoimmune nephropathy in this patient. Finally, there is sometimes a tendency in chromosomal deletions to attribute unusual findings to the deleted area. This paper, however, provides a great example of coexistence of two genetic mechanisms in the same patient resulting into two phenotypes that can underlie atypical presentations of primary immunodeficiency.

## 6. Conclusions

We present an interesting patient with APECED and 4q deletion with autoimmune nephritis. Based on the fact that autoimmunity is a basic pathogenesis mechanism in APECED, our report presents more evidence of autoimmune nephropathy in APECED. The diagnostic approach to this case also depicts two unrelated genetic hits causing two different phenotypes in the same patient.

## Supplementary Material

The short description for the supplementary table is as follows: “The gene list within the deleted region based on NCBI Build 37.1 for *Homo sapiens*”.

## Figures and Tables

**Table 1:** *AIRE *PCR and sequencing primers.

Primers	Sequences (5′–3′)	Tm
1 Forward	cgtggtcgcgggggtataaca	63°C
1 Reverse	tatccctggctcacagggcct	
2 Forward	ccccagccccaccctcaacac	65°C
2 Reverse	ccctttgcctcttaggatcca	
3 Forward	agctggactggaaccggagtg	60°C
3 Reverse	aaatgagacccgcccgcctac	
4 Forward	tgaagtaggcgggcgggtctc	65°C
4 Reverse	gacacaccaggccagcacgtc	
5 Forward	cacttgggtgcacacacgaac	60°C
5 Reverse	ttgcagaaggtcccccgacag	
6/7 Forward	tccccggccccagactcgac	65°C
6/7 Reverse	tcccagtggatccttgacctc	
8 Forward	aggaagggttcatgtggttgg	65°C
8 Reverse	cagcctgggatgagcttggac	
9 Forward	ccgttcctccttgccgtctc	55°C
9 Reverse	gccgttatcaatgctcatag	
10 Forward	agggtcccagcagtcactg	65°C
10 Reverse	ccctgtgcctcccggagcc	
11 Forward	agagaggtgcgggcgccagg	65°C
11 Reverse	tccccgccgaccacgctcac	
12 Forward	ccccacaccccataccccgga	65°C
12 Reverse	caggactctcaggctcatgc	
13 Forward	tggccctggtggtgcttgtc	65°C
13 Reverse	agcagtgggggccggcagtc	
14 Forward	tttgatggaatacggtgaag	60°C
14 Reverse	gcagatggtggtggcaatgg	
