# Supplementary material for: Renal Failure Associated with APECED and Terminal 4q Deletion: Evidence of Autoimmune Nephropathy
Source: Clin Dev Immunol. 2010 Dec 14;2010:586342. doi: 10.1155/2010/586342 (PMC3010696; doi:10.1155/2010/586342)
Supplement: Supplementary file 1 — The short description for the supplementary table is as follows: “The gene list within the deleted region based on NCBI Build 37.1 for Homo sapiens”. [file 586342.f1.pdf]

NCBI MAPVIEWER BUILD 37.1 (The current/Latest build)

Region Displayed: 177,600K-191,200K bp

Genes in Region: 90

Chr4 Start	Chr4 Stop	Symbol	O	OMIM	HGNC	Evidence	Cyto	Description
177604694	177713895	VEGFC	-	OMIM	HGNC	best RefSeq	4q34.1-q34.3	vascular endothelial growth factor C
178230991	178284092	NEIL3	+	OMIM	HGNC	best RefSeq	4	nei endonuclease VIII-like 3 (E. coli)
178351924	178363591	AGA	-	OMIM	HGNC	best RefSeq	4q32-q33	aspartylglucosaminidase
178649907	178911669	LOC285501	+			best RefSeq	4	hypothetical protein LOC285501
179207825	179208518	RPL19P8	+		HGNC	best RefSeq	4	ribosomal protein L19 pseudogene 8
179990579	179990770	LOC100288304	+			protein	4	similar to cytochrome c oxidase subunit II
181494617	181494906	LOC100288337	-			protein	4	hypothetical LOC100288337
182443813	182444574	LOC132386	+			best RefSeq	4	tumor-associated calcium signal transducer 1 pseudogene
182525584	182526434	LOC100288373	+			mRNA	4	hypothetical protein LOC100288373
182826942	182827602	LOC100128118	-			protein	4	similar to cyclin H
183060159	183065668	MGC45800	-			best RefSeq	4	hypothetical LOC90768
183090446	183090531	MIR1268	+			external	4q34.3	microRNA 1268
183245137	183724177	ODZ3	+	OMIM	HGNC	best RefSeq	4	odz, odd Oz/ten-m homolog 3 (Drosophila)
183811244	183838630	DCTD	-	OMIM	HGNC	best RefSeq	4	dCMP deaminase
183949753	183950224	LOC100132090	-			best RefSeq	4	survival motor neuron pseudogene
183958818	183961272	FAM92A3	+		HGNC	best RefSeq	4q35.1	family with sequence similarity 92, member A3
184018170	184020352	LOC100128823	+			mRNA	4	hypothetical LOC100128823
184018174	184020352	C4orf38	-		HGNC	best RefSeq	4	chromosome 4 open reading frame 38
184020463	184241930	WWC2	+		HGNC	best RefSeq	4	WW and C2 domain containing 2
184239220	184241927	CLDN22	-		HGNC	best RefSeq	4	claudin 22
184242917	184243579	CLDN24	-			protein	4	claudin-24-like
184272251	184302858	LOC100129666	+			protein	4	similar to chimerin (chimaerin) 1
184340035	184340958	LOC100127981	-			protein	4	similar to fibrillarin
184362513	184366612	LOC100131811	-			mRNA	4	hypothetical LOC100131811
184365789	184369049	CDKN2AIP	+		HGNC	best RefSeq	4	CDKN2A interacting protein
184404506	184405569	LOC389246	+			best RefSeq	4	VTI1B pseudogene
184426220	184432249	ING2	+	OMIM	HGNC	best RefSeq	4q35.1	inhibitor of growth family, member 2
184560789	184580331	RWDD4A	-		HGNC	best RefSeq	4	RWD domain containing 4A
184580446	184634745	C4orf41	+		HGNC	best RefSeq	4	chromosome 4 open reading frame 41
184826509	184938875	STOX2	+		HGNC	best RefSeq	4	storkhead box 2
185009859	185139114	ENPP6	-		HGNC	best RefSeq	4	ectonucleotide pyrophosphatase/phosphodiesterase 6
185175688	185176474	RPL6P16	+		HGNC	best RefSeq	4	ribosomal protein L6 pseudogene 16
185220282	185238554	LOC391722	-			protein	4	similar to myosin regulatory light chain MRCL2
185262843	185269065	LOC728175	-			mRNA	4	similar to MGC24125 protein
185308876	185395726	IRF2	-	OMIM	HGNC	best RefSeq	4q34.1-q35.1	interferon regulatory factor 2
185480329	185480937	LOC100129245	-			protein	4	similar to nucleolar protein family A, member 1
185548850	185570629	CASP3	-	OMIM	HGNC	best RefSeq	4q34	caspase 3, apoptosis-related cysteine peptidase
185570767	185616112	CCDC111	+		HGNC	best RefSeq	4	coiled-coil domain containing 111
185615236	185655286	MLF1IP	-	OMIM	HGNC	best RefSeq	4	MLF1 interacting protein
185676749	185747215	ACSL1	-	OMIM	HGNC	best RefSeq	4q34-q35	acyl-CoA synthetase long-chain family member 1
185719450	185720200	SLED1	-			best RefSeq	4	RTFV9368
185940083	185941926	HELT	+		HGNC	best RefSeq	4	HES/HEY-like transcription factor
186064398	186068425	SLC25A4	+	OMIM	HGNC	best RefSeq	4q35	solute carrier family 25 (mitochondrial carrier; adenine nucleotide translocator), member 4
186080819	186125182	KIAA1430	-		HGNC	best RefSeq	4	KIAA1430
186131284	186285120	SNX25	+		HGNC	best RefSeq	4	sorting nexin 25
186285032	186300152	LRP2BP	-		HGNC	best RefSeq	4	LRP2 binding protein
186317840	186321390	ANKRD37	+		HGNC	best RefSeq	4	ankyrin repeat domain 37
186321443	186347068	UFSP2	-	OMIM	HGNC	best RefSeq	4	UFM1-specific peptidase 2
186346203	186348049	LOC100288644	+			mRNA	4	hypothetical protein LOC100288644
186350545	186370821	C4orf47	+		HGNC	best RefSeq	4	chromosome 4 open reading frame 47
186366336	186392913	CCDC110	-	OMIM	HGNC	best RefSeq	4	coiled-coil domain containing 110
186392591	186393417	LOC100131727	+			mRNA	4	hypothetical LOC100131727
186421814	186422626	LOC100131683	-			mRNA	4	hypothetical protein LOC100131683
186422851	186456712	PDLIM3	-	OMIM	HGNC	best RefSeq	4q35	PDZ and LIM domain 3

186506598	186877870	SORBS2	-		HGNC	best RefSeq	4	sorbin and SH3 domain containing 2
186990309	187006252	TLR3	+	OMIM	HGNC	best RefSeq	4q35	toll-like receptor 3
187065995	187093817	FAM149A	+		HGNC	best RefSeq	4	family with sequence similarity 149, member A
187110186	187113275	FLJ38576	-			mRNA	4	hypothetical LOC651430
187112674	187134617	CYP4V2	+	OMIM	HGNC	best RefSeq	4	cytochrome P450, family 4, subfamily V, polypeptide 2
187148672	187179625	KLKB1	+	OMIM	HGNC	best RefSeq	4q34-q35	kallikrein B, plasma (Fletcher factor) 1
187187118	187210835	F11	+	OMIM	HGNC	best RefSeq	4q35	coagulation factor XI
187249609	187250834	SLC25A5P6	-		HGNC	best RefSeq	4	solute carrier family 25 (mitochondrial carrier; adenine nucleotide translocator), member 5 pseudogene 6
187454809	187476537	MTNR1A	-	OMIM	HGNC	best RefSeq	4q35.1	melatonin receptor 1A
187508937	187644987	FAT1	-	OMIM	HGNC	best RefSeq	4q35	FAT tumor suppressor homolog 1 (Drosophila)
187543069	187544347	LOC100286957	+			mRNA	4	hypothetical protein LOC100286957
187821893	187821995	MRPS36P2	+		HGNC	best RefSeq	4q35.1	mitochondrial ribosomal protein S36 pseudogene 2
188667288	188669359	LOC644325	-			protein	4	similar to a disintegrin and metalloprotease domain 25
188891427	188892438	LOC389249	+			protein	4	similar to peroxiredoxin 6
188916925	188926199	ZFP42	+		HGNC	best RefSeq	4	zinc finger protein 42 homolog (mouse)
189012427	189026408	TRIML2	-		HGNC	best RefSeq	4	tripartite motif family-like 2
189027712	189028068	FAUP3	+		HGNC	best RefSeq	4q35.2	FBR-MuSV-associated ubiquitously expressed (fox derived) pseudogene 3
189060598	189068649	TRIML1	+		HGNC	best RefSeq	4	tripartite motif family-like 1
189270472	189271201	RPL7AP27	-		HGNC	best RefSeq	4	ribosomal protein L7a pseudogene 27
189308625	189315164	LOC644491	-			protein	4	similar to NMDA receptor regulated 2
189659525	189662227	LOC285442	+			best RefSeq	4	mitochondrial translational release factor 1-like pseudogene
190394060	190396545	HSP90AA4P	+		HGNC	best RefSeq	4q35.2	heat shock protein 90kDa alpha (cytosolic), class A member 4 (pseudogene)
190861974	190884359	FRG1	+	OMIM	HGNC	best RefSeq	4q35	FSHD region gene 1
190903678	190906026	TUBB4Q	-		HGNC	best RefSeq	4q35	tubulin, beta polypeptide 4, member Q
190940255	190945506	DUX4C	-			protein	4q35.2	double homeobox 4c
190945522	190948412	FRG2	-	OMIM	HGNC	best RefSeq	4q35	FSHD region gene 2
190959056	190959857	LOC644881	-			protein	4	similar to chemerin preproprotein
190962073	190964569	LOC100132380	+			protein	4	similar to Angiogenic factor with G patch and FHA domains 1
190987701	190990406	LOC100287020	+			mRNA	4	similar to facioscapulohumeral muscular dystrophy
190992093	190993703	LOC653543	+			best RefSeq	4	double homeobox, 4-like
190995386	190996996	LOC653544	+			best RefSeq	4	double homeobox, 4-like
190998679	191000289	LOC653545	+			best RefSeq	4	double homeobox, 4-like
191001979	191003583	LOC441056	+			mRNA	4	double homeobox, 4-like
191005267	191006883	DUX4	+	OMIM	HGNC	best RefSeq	4q35	double homeobox, 4
191008566	191010176	LOC653548	+			mRNA	4	double homeobox, 4-like
191011866	191013476	LOC728410	+			best RefSeq	4	double homeobox, 4-like
